# Primary gastric non-Hodgkin's lymphoma in Chinese patients: clinical characteristics and prognostic factors

**DOI:** 10.1186/1471-2407-10-358

**Published:** 2010-07-06

**Authors:** JiaJia Huang, WenQi Jiang, RuiHua Xu, HuiQiang Huang, Yue Lv, ZhongJun Xia, XiaoFei Sun, ZhongZhen Guan, TongYu Lin, ZhiMing Li

**Affiliations:** 1 State Key Laboratory of Oncology in Southern China, Sun Yat-sen University, Guangzhou, Guangdong, China; 2 Department of Medical Oncology, Cancer Center, Sun Yat-sen University, Guangzhou, Guangdong, China; 3 Department of Hematology & Oncology, Cancer Center, Sun Yat-sen University, Guangzhou, Guangdong, China; 4 Department of Pediatic Oncology, Cancer Center, Sun Yat-sen University, Guangzhou, Guangdong, China

## Abstract

**Background:**

Optimal management and outcome of primary gastric lymphoma (PGL) have not been well defined in the rituximab era. This study aimed to analyze the clinical characteristics, prognostic factors, and roles of different treatment modalities in Chinese patients with PGL.

**Methods:**

The clinicopathological features of 83 Chinese patients with PGL were retrospectively reviewed. Staging was performed according to the Lugano staging system for gastrointestinal non-Hodgkin's lymphoma.

**Results:**

The predominant pathologic subtype among Chinese patients with PGL in our study was diffuse large B cell lymphoma (DLBCL), followed by mucosa-associated lymphoid tissue (MALT) lymphoma. Among the 57 patients with gastric DLBCL, 20 patients (35.1%) were classified as the germinal center B cell-like (GCB) subtype and 37 patients (64.9%) as the non-GCB subtype. The 83 patients had a five-year overall survival (OS) and event-free survival (EFS) of 52% and 59%, respectively. Cox regression analysis showed that stage-modified international prognostic index (IPI) and performance status (PS) were independent predictors of survival. In the 67 B-cell lymphoma patients who received chemotherapy, 36 patients treated with rituximab (at least 3 cycles) had a mean OS of 72 months (95% CI 62-81) versus 62 months (95% CI 47-76) for patients without rituximab treatment (P = 0.021).

**Conclusion:**

The proportion of Chinese gastric DLBCL cases with non-GCB subtype was higher than the GCB subtype. Stage-modified IPI and PS were effective prognostic factors in Chinese patients with PGL. Our data suggested that primary gastric B-cell lymphoma might have an improved outcome with rituximab in addition to chemotherapy. More studies are necessary, preferentially large prospective randomized clinical trials to obtain more information on the impact of the rituximab in the primary gastric B-cell lymphoma.

## Background

Primary gastric lymphoma (PGL) originates in the stomach, with or without perigastric and/or abdominal lymph node involvement [1]. PGL is an uncommon tumor, accounting for less than 15% of gastric malignancies and about 2% of all lymphomas [2]. However, PGL is the most common extranodal lymphoma, representing 30%-40% of all extranodal lymphomas and 60%-75% of all gastrointestinal lymphomas [3-6]. The incidence of PGL is progressively increasing.

The main histological subtypes of PGL (more than 90% of cases) are diffuse large B-cell lymphoma (DLBCL) and marginal zone B-cell lymphoma of the mucosa-associated lymphoid tissue (MALT) [7,8]. Helicobacter pylori infection has been implicated in the pathogenesis and treatment of gastric MALT lymphoma, but its role in gastric DLBCL is uncertain[9,10]. Various therapeutic aspects for primary gastric lymphomas, including antibiotic therapy, rituximab therapy, combining chemotherapy with radiotherapy or occasionally resection, are still controversial and several questions remain unanswered. In the past, gastrectomy was the front-line treatment in patients with PGL. However, recent clinical trial results supported that organ preservation with chemotherapy combined with radiation could yield equal results to surgery combined with radiation in PGL patients [11]. Among others, clinical information of PGL warrants better clarification. Thus, we decided to contribute to this field by investigating the clinical characteristics, prognostic factors, and the roles of different treatment modalities of PGL in the rituximab era.

## Methods

### Patients and Staging

We carried out a retrospective study of 83 PGL patients diagnosed at the Sun Yat-sen University Cancer Center, China from January 2001 to June 2008. The study was approved by the Institutional Review Board (IRB) of Sun Yat-Sen University Cancer Center. All the cases satisfied the PGL diagnosis criteria defined by Lewin et al. [12], and were identified based on the World Health Organization (WHO) classification of Tumor of Hematopoietic and Lymphoid Tissues [7]. All of the patients were negative in the serologic detection of Human immunodeficiency virus (HIV).

All patients were staged according to the Lugano staging system for gastrointestinal non-Hodgkin's lymphoma [8]. The diagnostic workup included the patients' history, performance status according to the Eastern Cooperative Oncology Group (ECOG) scale, physical examination, baseline endoscopy or barium meal examination, gastric mucosal biopsies or gastrectomy, complete blood cell count, biochemical profile, measurement of serum lactate dehydrogenase (LDH), computed tomography scans of the thorax, abdomen and pelvic cavity, as well as bone marrow aspiration and biopsy. We defined bulky disease as any mass of 10 cm or more in maximal diameter. Low hemoglobin was defined as < 120 g/L, low albumin as < 35 g/L, and high LDH as > 245 U/L.

### Imunohistochemical Study and Research of H. Pylori

Formalin-fixed paraffin-embedded tissues obtained from patients diagnosed with DLBCL were analyzed for immunoreactivity towards CD20 (clone L26, DAKO, Glostrup, Denmark), BCL6 (clone P1F6, Novocastra, Newcastle, UK), CD10 (clone 56C6, Novocastra, Newcastle, UK) and MUM1/IRF4 (polyclonal, Santa Cruz Biotechnology, Santa Cruz, CA, USA). We analyzed the tissues for immunoreactivity towards CD10, bcl-6, or MUM1 according to the immunophenotypic profile criteria of DLBCL described by Hans et al. [13]. Cases where 30% or more of the tumor cells were immunoreactive with a given antibody were considered positive for that antigen [13]. The immunohistochemical review was performed independently by 2 histopathologists. Patients were considered H. pylori positive if either the serology or histology was positive for H. pylori.

### Stage-modified International Prognositic Index (IPI)

Stage-modified IPI was designed according to the IPI (international prognostic index) in which the original Ann Arbor stage II was substituted by Lugano staging system for gastrointestinal non-Hodgkin's lymphoma [14].

### Response Criteria

We used imaging studies and endoscopic examination in order to perform response evaluation. Response criteria were defined according to the International Working Group Recommendations [15]. Complete remission (CR) was defined as the complete disappearance of all physical and radiological evidence of disease for at least four weeks. An endoscopic evaluation and biopsies of stomach should be carried out to confirm a CR. Additionally, patients not in CR at the end of treatment were considered treatment failures. Overall survival (OS) was measured from the date of diagnosis until the date of death due to any cause or the date of final follow-up in the survivors. Event-free survival (EFS) was calculated from the date of diagnosis to the date of treatment failure, relapse, evidence of disease progression or death due to any cause.

### Statistical Analysis

Primary endpoints of our analysis were OS and EFS. Survival curves and the univariate analysis were analyzed by the Kaplan-Meier method. The prognostic value of different variables for clinical outcome was estimated by multivariate analysis using the Cox regression model. Clinical parameters with corresponding *P*-value of less than 0.05 in univariate analysis were included in multivariate analysis. A 2-tailed P value < 0.05 assessed by the log-rank test was considered statistically significant. SPSS 16.0 for Window Software was used for all statistical analyses.

## Results

### Clinical and Histological Features

Patients' characteristics are detailed in Table 1. Of the 83 patients recruited into the study, 45 were male and 38 were female with a median age of 52 years (range 15-81 years). The most common complaint at presentation was epigastric pain. Thirty cases (36%) of the patients presented with melena and fifteen cases (18%) with nausea/vomiting. Hematemesis and ileus were uncommon at presentation. None of the patients experienced perforation prior to treatment. In our study, we found that the antrum was the most commonly involved site (53 cases, 64%), followed by the corpus (44 cases, 53%). Two cases were diagnosed with stump involvement. Both had a history of gastric ulcer and underwent subtotal gastrectomy 20 and 22 years prior to study participation, respectively.

**Table 1:** Baseline Characteristics of 83 Chinese Patients with PGL

Characteristics	Number of assessable patients (%)
**Age (years)**	
Median	52
Range	15-81
**Sex**	
Male	45 (54.2)
Female	38 (45.8)
Ratio, male to female	1.2
**Presenting Symptom^1^**	
Abdominal pain	69 (83.1)
Melena	30 (36.1)
Nausea or vomitting	15 (18.1)
Hematemesis	6 (7.2)
Ileus	1 (1.2)
Perforation	0
**B symptoms**	56 (67.5)
**Bulky Disease**	21 (25.3)
**Involvement within the stomach^2^**	
Cardia	13 (15.7)
Fundus	15 (18.1)
Corpus	44 (53.0)
Antrum	53 (63.9)
Pylorus	12 (14.5)
Stump	2 (2.4)
**Histopathology**	
B cell subtypes	75 (90.4)
Diffuse large B cell lymphoma	57 (68.7)
GCB subtype	20 (20/57, 35.1)
Non-GCB subtype	37 (37/57, 64.9)
Ratio, GCB to Non-GCB	1:1.85
MALT lymphoma	14 (16.9)
Burkitt lymphoma	3 (3.6)
Mantle cell lymphoma	1 (1.2)
T cell subtypes	8 (9.6)
Peripheral T-cell lymphoma, unspecified	4 (4.8)
Anaplastic large cell lymphoma	3 (3.6)
Precursor T lymphoblastic lymphoma	1 (1.2)
H. pylori infection^3^	49 (49/61, 80.3)
Laboratories at first visit	
Leucocytopenia (< 4.0 × 10^9^/L)	7 (8.4)
Neutropenia (< 2.0 × 10^9^/L)	8 (9.6)
Lymphocytopenia (< 1.0 × 10^9^/L)	15 (18.1)
Low hemoglobin (< 120 g/L)	43 (51.8)
Low albumin (< 35 g/L)	24 (28.9)
Increased LDH (> 245 U/L)	40 (48.2)
Lugano Staging	
I	24 (28.9)
II1	5 (6.0)
II2	14 (16.9)
IIE	20 (24.1)
IV	20 (24.1)

Number total > 83, more than 1 symptom can be observed in the same patient.

Number total > 83, more than 1 involved site can be found in the same stomach.

61 of 83 cases received H. pylori detection.

According to the results of the immunophenotypic profile in DLBCL, we grouped the fifty-seven patients (69%) with DLBCL into the germinal center B-cell-like group (GCB) and the non-GCB group. We demonstrated that of the 57 patients with DLBCL, 20 patients (35%) were classified as the GCB subtype and 37 cases (65%) as the non-GCB subtype. The ratio of GCB to non-GCB phenotypes was about 1:1.85. MALT lymphoma accounted for 17% of all the patients studied (14 cases). Other B cell histological subtypes were found in 4 cases, including 3 cases of Burkitt's lymphoma and 1 case of mantle lymphoma. Eight cases (9.6%) were diagnosed as T-cell NHL, with 1 case of precursor T lymphoblastic lymphoma, 3 cases of anaplastic large cell lymphoma (ALCL), and 4 cases of unspecified peripheral T-cell lymphoma (U-PTL).

Among the 61 patients who were tested for H. pylori, there were 43 cases with DLBCL, 12 cases with MALT, and 6 cases with other pathologic subtypes. Forty-nine study patients (80%) tested positive for H. pylori. The DLBCL and MALT lymphoma groups comprised 79% (34/43) and 92% (11/12) of the H. pylori positive patients respectively.

Using the Lugano staging system, 24 patients (29%) presented at Stage I. Of the 19 patients (23%) who presented with local or distant nodal involvement, 5 were diagnosed with local lymph node involvement (stage II1), and 14 had distant abdominal nodal extension (stage II2). There were also 20 cases (24%) who were at stage IIE and 20 cases (24%) at stage IV with advanced disease. Table 2 represents the classification of patients into the adverse factors groups and three high-risk groups of stage-modified IPI.

**Table 2:** Patients' Characteristics According to Stage-modified IPI

Characteristics (adverse factors of all patients)	Number of patients (%)
Age > 60 years	24 (28.9)
Serum LDH > 245 U/L	40 (48.2)
ECOG Performance Status ≥ 2	9 (10.8)
Lugano stage ≥ II2	54 (65.1)
Extranodal site involvement (excluding stomach) ≥ 1	9 (10.8)

**Risk groups (number of risk factors)**	

Low risk group (0-1)	38 (45.8)
Intermediate risk group (2)	26 (31.3)
High risk group (3-5)	19 (22.9)

### Treatment Modalities

Table 3 shows the treatment modalities of all patients. Of the 28 patients who underwent surgery, 18 patients had radical surgery and 10 patients had palliative surgery. Four of these 28 cases underwent emergency surgery due to chemotherapy related complications, including perforation, acute upper gastrointestinal hemorrhage and intestinal obstruction. Of the two patients with surgical complications: one had reflux esophagitis and the other had stenosis of the stoma.

**Table 3:** Treatment Modalities of 83 Chinese Patients with PGL

Treatment modalities	Number of patients	Histological Subtypes (no. of patients)		Lugano Staging (no. of patients)	
		
		B-cell lymphoma (n = 75)	T-cell lymphoma (n = 8)	I-II1 (n = 29)	II2-IV (n = 54)
**Single therapy**	32	30	2	16	16
Surgery	1	1	0	0	1
Chemotherapy	29	27	2	14	15
H. pylori eradication	2	2	0	2	0
**Combined therapy**	45	40	5	13	32
Surgery + chemotherapy	23	20	3	6	17
Surgery + chemotherapy + H. pylori eradication	2	1	1	1	1
Chemotherapy + radiotherapy	14	13	1	3	11
Chemotherapy + radiotherapy + H. pylori eradication	4	4	0	3	1
Surgery + chemotherapy + radiotherapy	2	2	0	0	2
**Supportive care only**	6	5	1	2	4
**Treatment in both single and combined therapy**					
Surgery	28	24	4	7	21
Chemotherapy	74	67	7	27	47
Radiotherapy	20	19	1	6	14
H. pylori eradication	8	7	1	6	2

Of the 74 patients who received chemotherapy, 29 patients received only chemotherapy and 45 patients received chemotherapy combined with other treatment modalities. CHOP (cyclophosphamide, doxorubicin, vincristine and prednisone) or CHOP-like regimens were the most frequently used first-line treatment in 67 patients (49 patients with DLBCL, 12 patients with MALT lymphoma, 2 cases with Burkitt lymphoma, 1 case with mantle cell lymphoma, 1 case with precursor T lymphoblastic lymphoma, 1case with anaplastic large cell lymphoma and 1 case with peripheral T-cell lymphoma, unspecified). Other regimens included EPOCH (etoposide, prednisone, vincristine, doxorubicin and cyclophosphamide) (3 cases with DLBCL, 1 case with anaplastic large cell lymphoma and 1 case with peripheral T-cell lymphoma, unspecified), IMVP-16 (ifosfamide, methotrexate and etoposide) (1 case with peripheral T-cell lymphoma, unspecified) and GEMOX (gemcitabine and oxaliplatin) (1 case with peripheral T-cell lymphoma, unspecified). Thirty-six patients treated with rituximab for at least 3 cycles (ranged from 3 to 8 cycles), with a median of 5 cycles. Chemotherapy related complications such as acute upper gastrointestinal hemorrhage and intestinal obstruction, perforation and worsening of performance status (excluding hematological toxicity) were observed in 9 of 74 patients who received chemotherapy. There were 3 cases with acute bleeding, 1 case with ileus, 2 cases with acute bleeding and ileus, 2 cases with perforation, and 1 case with worsening of performance status. Four of these cases underwent emergency surgery due to acute bleeding, ileus or perforation. Two patients died due to acute upper gastrointestinal hemorrhage related to the treatment. Of the twenty patients (24%) who received radiotherapy, eighteen received it as adjuvant treatment followed by chemotherapy, while two patients had radiotherapy as palliative treatment after disease progression.

Two patients who were diagnosed as MALT lymphoma (stage I) and H. pylori positive were only treated for H. pylori. Both survived with complete tumor remission as evaluated by imaging studies and endoscopic examinations at their 41 and 43 month follow-up visits, respectively. Six of the of 83 patients (7.2%) patients received supportive care due to their poor performance status, and all of them died of tumor progression.

### Survival and prognostic factors

Twenty-seven of the 83 patients (32%) died in a median follow-up period of 48 months (range 2-95 months). Seventeen of these patients died of tumor progression, 2 died of chemotherapy related hemorrhage, 3 died of non-malignancies and 5 died of unknown causes.

**Figure 1:**
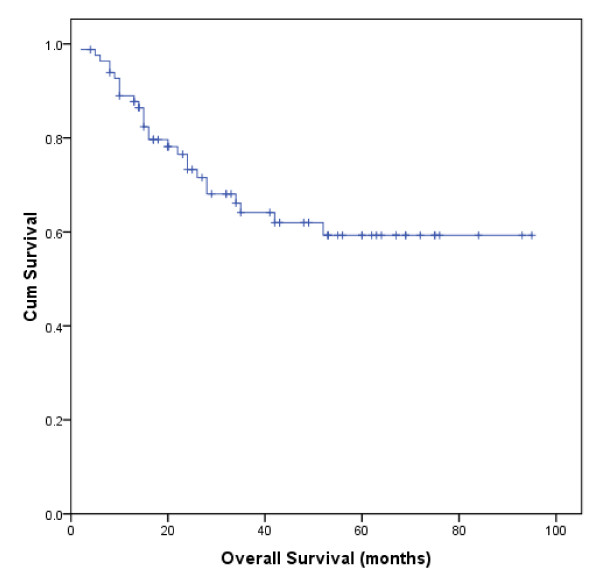
**Overall Survival of 83 Chinese Patients with PGL**.

An actuarial analysis showed that 1-year and 5-year estimates of overall survival were 89% and 59%, respectively, with a mean survival time (MST) of 65 months (95% confidence interval, CI 56-74) (Figure 1). In patients with poor performance status (PS ≥ 2), the mean survival time was 17 months (95% CI 5-29) versus 71 months (95% CI 62-80) for those with better performance status (PS = 0-1) (P < 0.0001). We also showed that stage-modified IPI was a good predictor of overall survival. Patients in the low risk group (stage-modified IPI = 0-1) had an MST of 87 months (95% CI 78-95) versus 44 months (95% CI 33-54) for those in the intermediate and high risk groups (stage-modified IPI = 2-5). In univariate analysis, advanced Lugano staging (≥ stage II2), elevated LDH levels, poor PS, stage-modified IPI ≥ 2, low albumin, bulky disease and lymphocytopenia were all related to shorter overall survival. In multivariate analysis of OS, PS (relative risk, RR = 8.02, 95% CI 3.11-20.71, P < 0.0001) and stage-modified IPI ≥ 2 (RR = 9.02, 95% CI 2.67-30.51, P < 0.0001) remained significant predictors. Table 4 summarizes a univariate and multivariate analysis of the factors considered as predictors of overall survival.

**Table 4:** Risk Factors Associated with OS in 83 Chinese Patients with PGL

	Univariate Analysis		Multivariate Analysis	
		
Variable	Mean survival of OS (months) (95%CI)	P value	RR (95%CI)	P value
Sex				
Female	67 (55,80)	0.622		
Male	52 (43,62)			
Age				
≤ 60 years	66 (55,76)	0.795		
> 60 years	44 (34,53)			
B symptoms				
No	67 (55,79)	0.186		
Yes	60 (49,71)			
LDH				
≤ 245 U/L	81 (70,91)	< 0.0001	Not significant	
> 245 U/L	45 (33,56)			
Extranodal involvement (excluding stomach)				
No	67 (58,76)	0.112		
Yes	37 (15,58)			
Lugano staging				
I-II1	88 (78,97)	< 0.0001	Not significant	
≥ II2	49 (39,59)			
ECOG performance status (PS)				
0-1	71 (62,80)	< 0.0001	8.08 (3.13, 20.89)	0.0001
≥ 2	17 (5,29)			
Histological subtypes^1^				
DLBCL	65 (54, 76)	0.098		
MALT	78 (61, 95)			
T-cell lymphomas	37 (17, 59)			
mIPI				
0-1	87 (78,95)	< 0.0001	8.49 (2.50, 28.85)	0.001
≥ 2	44 (33,54)			
Bulky disease				
No	73 (63,83)	0.004	Not significant	
Yes	42 (28,57)			
Leucocytopenia				
No	65 (56,75)	0.955		
Yes	39 (23,56)			
Neutropenia				
No	65 (55,74)	0.833		
Yes	40 (24,56)			
Lymphocytopenia				
No	68 (59,78)	0.023	Not significant	
Yes	42 (20,63)			
Anemia				
No	72 (58,82)	0.235		
Yes	60 (47,72)			
Albumin				
≥ 35 g/L	73 (64,83)	0.003	Not significant	
< 35 g/L	44 (29,58)			

^1^Patients with DLBCL (57 cases), MALT lymphoma (14 cases) and T-cell lymphomas (8 cases) were included for analysis, and other histological subtypes were excluded.

**Figure 2:**
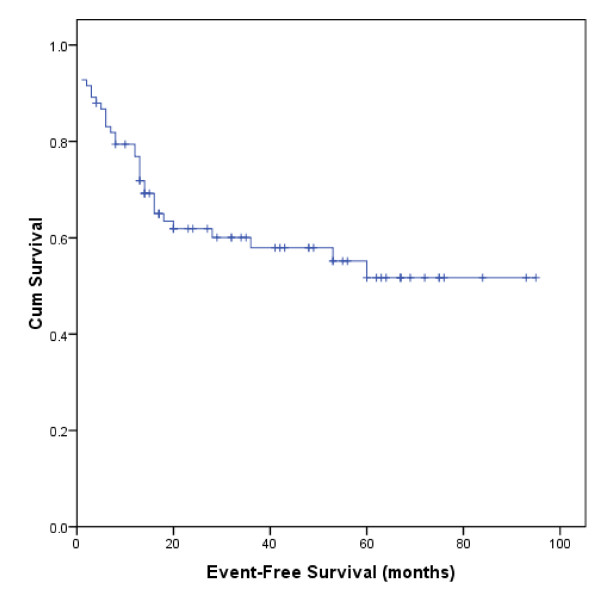
**Event-Free Survival of 83 Chinese Patients with PGL**.

Figure 2 demonstrates that the actuarial estimates of EFS at 1 and 5 year were 77% and 52%, respectively, with a median EFS of 57 months (95% CI 48-67). Possible predictive variables of EFS were also estimated by univariate and multivariate analysis, as shown in Table 5. We found that PS ≥ 2 (RR: 7.45, 95% CI 2.96-18.73, P < 0.0001), stage-modified IPI ≥ 2 (RR: 3.63, 95%CI 1.51-8.72, P = 0.004) and low hemoglobin (RR: 2.38, 95%CI 1.08-5.27, P = 0.032) were effective predictors of EFS in Cox multivariate analysis.

**Table 5:** Risk Factors Associated with EFS in 83 Chinese Patients with PGL

	Univariate Analysis		Multivariate Analysis	
		
Variable	Mean survival of EFS (month)(95%CI)	P value	RR (95%CI)	P value
Sex				
Female	59 (46,73)	0.697		
Male	46 (36,56)			
Age				
≤ 60 years	58 (47,69)	0.875		
> 60 years	41 (30, 52)			
B symptoms				
No	59 (45,72)	0.294		
Yes	53 (41,65)			
LDH				
≤ 245 U/L	70 (57,82)	0.001	Not significant	
> 245 U/L	39 (27,51)			
Extranodal involvement (excluding stomach)				
No	59 (49,69)	0.160		
Yes	32 (12,52)			
Lugano staging				
I-II1	82 (70,93)	< 0.0001	Not significant	
≥ II2	41 (30,51)			
ECOG performance status (PS)				
0-1	62 (53,72)	< 0.0001	7.45 (2.96, 18.73)	0.0001
≥ 2	11 (0,24)			
Histological subtypes^1^				
DLBCL	58 (47, 69)	0.261		
MALT	73 (54, 91)			
T-cell lymphomas	34 (13, 56)			
mIPI				
0-1	77 (65,89)	< 0.0001	3.63 (1.51, 8.72)	0.004
≥ 2	37 (26,48)			
Bulky disease				
No	66 (55,76)	0.001	Not significant	
Yes	30 (15,45)			
Leucocytopenia				
No	59 (49,69)	0.179		
Yes	24 (5,43)			
Neutropenia				
No	58 (49,68)	0.372		
Yes	29 (11,47)			
Lymphocytopenia				
No	63 (53,73)	0.001	Not significant	
Yes	27 (8,46)			
Anemia				
No	70 (58,82)	0.007	2.38 (1.08, 5.27)	0.032
Yes	45 (33,58)			
Albumin				
≥ 35 g/L	67 (56,77)	0.001		
< 35 g/L	33 (19,48)		Not significant	

We also evaluated the impact of surgery and radiotherapy in the management of PGL. We found that the mean overall survival for patients treated with chemotherapy alone was 58 months (95% CI 44-72). OS was 67 months (95% CI 52-82) in patients treated with chemotherapy combined with surgery and 73 months (95% CI 59-87) in those treated with chemotherapy combined with radiotherapy. However, we found no statistically significant difference between these three groups (P > 0.05). Of the 67 B-cell lymphoma patients who received chemotherapy, 36 patients treated with rituximab concurrently with or followed by chemotherapy (Table 6). Patients with rituximab therapy had a mean survival time of 72 months (95% CI 62-81) versus 62 months (95% CI 47-76) in those without rituximab therapy (P = 0.021).

**Table 6:** Histological subtypes and Lugano staging of 67 patients with B-cell lymphoma who received chemotherapy with or without rituximab

	Rituximab therapy (no. of patients) (n = 36)	No rituximab therapy (no. of patients) (n = 31)
Histological subtypes		
DLBCL	30	22
MALT lymphoma	4	8
Burkitt lymphoma	1	1
Mantle cell lymphoma	1	0
Lugano Staging		
I-II1	14	12
II2-IV	22	19

## Discussion

Primary gastric non-Hodgkin's lymphoma comprises about 4-20% of all non-Hodgkin lymphomas [6,11,16]. PGL is also the most common extranodal NHL in the Hong Kong Chinese population, accounting for up to 80% of all extranodal NHL. This is higher than the numbers reported in western countries [17]. Our analysis of 57 DLBCL patients among the PGL cases showed that the ratio of the GCB to non-GCB subtype was 1:1.85 and is similar to a previous study from Hong Kong. In the Hong Kong study, 32 patients with DLBCL were comprised of 10 (31.2%) GCB and 22 (68.8%) non-GCB phenotypes [17]. However, in a study of 141 patients with primary gastric DLBCL from Japan, Italy and France, the ratio of GCB to non-GCB phenotypes was about 1.04:1 [18]. The ratio of GCB to non-GCB subtypes in Chinese patients with DLBCL of PGL has not been extensively investigated and the sample sizes analyzed so far, including in our study, have been small [17,18]. It will be interesting to explore differences in the distribution of pathology subtypes between Chinese and non-Chinese patients with PGL.

In the 69 patients who received H. pylori detection, the incidence of H. pylori infection for cases with DLBCL was lower than those with MALT lymphoma: 79% versus 92%, respectively. H. pylori is considered linked to the development of MALT lymphoma, although its role in DLBCL is a matter of debate [9,10,19].

The value of possible prognostic factors such as B symptoms, LDH levels and PS remains controversial. No risk factors have been clearly identified in PGL so far and the International Prognostic Index (IPI) is a commonly used predictive model for aggressive non-Hodgkin's lymphoma. However, the role of IPI in PGL is doubtful [1,20,21]. Since the modified-stage IPI proposed by S. Cortelazzo et al. was applied in a limited manner to patients with localized primary gastric DLBCL (stage I-IIE) [14], the predictive value of stage-modified IPI in advanced stage or other subtypes lymphoma remains unexplored. In our study, we analyzed the clinical characteristics of Chinese patients with different subtypes (predominantly DLBCL) of PGL presenting at both limited and advanced Lugano stages. We found that performance status and stage-modified IPI could effectively predict prognosis of PGL.

In recent years, surgery has gradually been replaced by radiotherapy in the treatment of PGL. However, surgery has been seen to benefit patients who present with hemorrhage, perforation or ileus [11,22,23]. We found no significant difference when we compared the overall survival of patients treated either with chemotherapy alone or with combination therapy (chemotherapy combined with radiation or with surgery). Approximately two thirds of the patients recruited in our study presented at an advanced Lugano stage (≥ II2). We propose that surgery or radiotherapy work by improving overall survival in early stage PGL patients rather than advanced cases. This might explain why we saw no significant differences between different treatment modalities in our analysis. The small sample size analyzed in our study could be a possible reason for the lack of OS difference between the different treatment modalities.

Rituximab, a chimeric anti-CD20 antibody, was found to be effective in terms of improving survival rate as well as eliciting an effective clinical response when used in combination with conventional chemotherapy for various subtypes of B-cell lymphoma patients [24-26]. As far as we know, there were no previous studies directly compared immunotherapy-chemotherapy with chemotherapy alone in PGL. The study of Wohrer et al. showed that rituximab in addition to chemotherapy was promising in early-stage gastric DLBCL [27]. A retrospective study of 75 patients in Japan also displayed an excellent result in gastric DLBCL [28]. However, a phase II clinical trial showed that the addition of rituximab to standard chemotherapy did not improve the outcome in early-stage PGL [29]. The role of rituximab in PGL remained controversial.

In this series, our data suggested that rituximab might improve the efficacy of chemotherapy in patients with primary gastric B-cell lymphoma. It was speculated that since conventional chemotherapy showed excellent results for early-stage PGL, adding rituximab may only have added limited incremental value [29]. The situation was quite different in our report. Of the 36 patients who received rituximab therapy in our study, 22 patients (61%) had advanced disease and carried a worse prognosis. The effect of rituximab could well display in patients with advanced stage. The effect of rituximab combined with chemotherapy in B-cell PGL remains controversial [26,27,30]. It will be very informative to study the exact role of rituximab in patients with PGL in large prospective randomized clinical trials.

## Conclusion

In this study, we found a much higher proportion of the non-GCB subtype among Chinese gastric DLBCL patients than the GCB subtype. Performance status and stage-modified IPI were effective predictors of prognosis in Chinese patients with primary gastric lymphoma at all stages. Our data suggested that primary gastric B-cell lymphoma might have better outcome with rituximab treatment in addition to chemotherapy. More studies are necessary, preferentially large prospective randomized clinical trials to obtain more information on the impact of the rituximab in the primary gastric B-cell lymphoma.

## Competing interests

There is no conflict of interest that could be perceived as prejudicing the impartiality of the study reported. This work is supported by National Natural Scientific Research Fund of China (Project Number: 30400589) and Key Projects in the National Science & Technology Pillar Program During the Eleventh Five-Year Plan Period of China.

## Authors' contributions

JJH and TYL carried out the conception and design of the study. ZML and WQJ participated in the design and administrative support for the study. All authors participated in the provision of patients. JJH carried out the collection and assembly of data. JJH and ZML participated in the data analysis and interpretation of the study, drafted the manuscript. All authors read and approved the final manuscript.

## Pre-publication history

The pre-publication history for this paper can be accessed here:

http://www.biomedcentral.com/1471-2407/10/358/prepub
